# Smoking cessation in dental setting: a narrative review on dental professionals’ attitude, preparedness, practices and barriers

**DOI:** 10.3389/froh.2023.1266467

**Published:** 2023-09-21

**Authors:** H. L. Chan, Alice K. Y. Chan, C. H. Chu, Y. C. Tsang

**Affiliations:** Faculty of Dentistry, The University of Hong Kong, The Prince Philip Dental Hospital, Sai Ying Pun, Hong Kong SAR, China

**Keywords:** smoking cessation, dental practice, smoking cessation advice, behavioural modification, smoking cessation challenge

## Abstract

Integration of smoking cessation program into routine oral health care has been advocated by World Health Organization since it brings extensive benefits to oral health. By tobacco cessation, patients are less prone to progression of periodontal disease, have less future tooth loss, have reduced risks of oral mucosal lesions and head and neck cancers. Evidence indicates that dentists are in a favorable position to deliver effective smoking cessation advice to improve patients’ oral health. This article aims to present the current situation of smoking cessation in dental setting, including dental management of smoking patients, perceptions of dentists and dental students towards smoking cessation, challenges dental professionals face when carrying out cessation interventions. Patients’ perspectives are also evaluated to provide a clearer picture of smoking cessation practice in the dental field. Review of past surveys show most patients welcome smoking cessation advice from dental practitioners. Meanwhile dentists may have wrong assumption that patients would disapprove them if they advise patient to quit smoking. On top of that, main obstacles identified are lack of training, inadequate treatment time and insufficient knowledge towards smoking cessation guidelines and referral routes. With regard to the potential barriers, evidence demonstrates that more trainings on smoking cessation strategies are needed. Future research in this aspect is also indicated to further foster the practice of smoking cessation counselling in dental setting.

## 1. Introduction

Smoking is well known as one of the causes of various non-communicable diseases. According to World Health Organization (WHO), 22.3% of the global population used tobacco in 2020. Tobacco alone kills more than 8 million people per year and total healthcare cost of smoking-attributable diseases reaches US$422 billion (1), posing heavy burden on our public health sector. It is evident that tobacco use, in form of conventional cigarettes and other forms of smoked tobacco (e.g., cigars, pipe, bidis, water pipes), smokeless tobacco products and e-cigarettes, is detrimental to oral health (2). Reversely, smoking cessation has been proved to be beneficial to oral health. According to the WHO, tobacco or smoking cessation is defined as self-reported continuous or point abstinence and biomedical validation is usually not required (2). The well-documented benefits include reduced risk of tooth loss, lower incidence and slower progression of periodontitis, lower incidence of oral mucosal lesions including leukoplakia and other pre-malignant lesions, and reversed risk of head and neck cancer (2–5).

Since smoking cessation brings substantial health benefits, global joint effort has been made to reduce the smoking prevalence in the world. In particular, WHO suggests the integration of smoking cessation service with primary health care system, with the emphasis on oral health professionals who have the greatest potential to promote reduction in tobacco use (2). As primary healthcare provider, dentists are able to approach a large pool of patients and for instance, 50% of adult smokers in USA visit a dentist each year (6). Dentists are also able to make personalized feedback to relate oral examination to the impact of tobacco smoking (7).

Thus, dentists are described as ‘in a unique position to deliver smoking cessation counselling.’ and dental setting is often considered ‘an excellent place for smoking cessation’ (6). Smoking cessation strategies that can be offered by dental professionals are brief intervention using 5As model (Ask, Advise, Assess, Assist and Arrange), behaviour counselling, nicotine replacement therapy (NRT), prescription of cessation drugs (e.g., varenicline and bupropion) (7–10). Each of them is considered safe and effective treatment according to 2020 Surgeon General’s report on smoking cessation recommendation (11). According to Article 14 of the WHO Framework Convention on Tobacco Control, dental practitioner should start with and at least carry out brief intervention with smoking patients. With a 3 min brief intervention, quit rate could be increased by 30% (12). Considering the nicotine dependence level of the patient, pharmacological treatment can be introduced in subsequent appointments (10). Two Cochrane reviews (13, 14) supported that behavior intervention and pharmacological treatment can improve the cessation efficacy. When used in combination, it could increase the quit rate by 7% (9).

Given the extensive evidence and academic support, it is crucial to evaluate the current situation of smoking cessation in dental setting to further help patients quit tobacco. Many cross-sectional surveys and questionnaires were conducted to assess dentists’ smoking cessation practices in different regions. However, most of them do not have a standardized method of assessment leading to scattered results. This article aims to provide a summary on dental professionals’ attitude towards smoking cessation, their preparedness in carrying out smoking cessation, and their usual practice of smoking cessation in dental setting. Patients’ attitude towards smoking cessation counselling in dental setting is also evaluated. Smoking cessation strategies covered in this article include (i) brief intervention (i.e., 5As), (ii) pharmacological treatment (i.e., nicotine replacement therapy and cessation medications).

## 2. Literature search

Literature search was performed in the PubMed, Cochrane Review and Google Scholar databases. Various keywords and their combination were used: ’smoking cessation’, ‘tobacco cessation’, ‘attitude’, ‘preparedness’, ‘practices’, ‘barriers’ etc. Articles were selected based on the following inclusion criteria: (1) published in English; (2) studies on smoking cessation by dentists, dental specialists and dental students; (3) studies assessing attitude, preparedness, practices and/or barriers of carrying out smoking cessation; (4) cross-sectional studies and (5) studies from 2000 to 2022. Excluded studies were (1) review articles; (2) experimental studies; (3) studies only on medical doctors and professionals and (4) articles only assessing attitude towards smoking amd smoking cessation. After literature search, 18 articles in total were included in this narrative review.

## 3. Dental professionals’ attitude in smoking cessation

Studies had various questions to assess dentists’ and dental students’ attitude on their role and responsibility in smoking cessation (15–27). Most commonly used questions were (1) ‘Is smoking cessation within the scope of dental practice?’, (2) ‘Does a dental practitioner have the role or responsibility to do smoking cessation?’, (3) ‘Is a dental practitioner/ dental team important in smoking cessation?’, (4) ‘Is dental clinic an appropriate place to give smoking cessation?’. Overall, studies have shown a positive and favourable attitude from dentists and dental students (15–27). Surveys demonstrated 70% to 96% of respondents believing smoking cessation in dental practice is important. Respondents supported that smoking cessation should be regarded as part of the dental treatment and patient management (19, 21, 22, 26).They showed willingness to provide smoking cessation service and advice to patient (24, 28). It was also reported that dental students consider themselves as a role model to patients (21), showing a sense of professional responsibility. In addition to a general statement, a few studies assessed whether a specific cessation strategy is within the scope of dental practice (22, 26). Anders et al. (26) reported fewer students supported the use of nicotine gum (61%) and patches (55%) are within the scope of dentistry; Ford et al. (22) reported more dentists agreed that it is appropriate to ask patient smoking status (94%) and explain oral risks associated with smoking (96.9%), than advising patient to completely quit (82%) or discuss the use of NRT (50%). Alongside with the overall positive perception, there were small portions showing negative concerns over smoking cessation. Some dentists rated smoking cessation as less important item than ordinary dental treatments (19, 20). Negative impacts on clinical practice included upsetting patient-dentist relationship (24), cessation counselling would be a waste of time (19, 20).

## 4. Dental professionals’ confidence, knowledge and preparedness in offering smoking cessation

Most studies assessed dentists’ and dental students’ self-perception of confidence and knowledge towards smoking cessation (15–33). Majority of dentists and dental students self-claimed that they were not confident and not knowledgeable in assisting patient to quit smoking, with rates from 14% to 61% feeling knowledgeable or prepared in smoking cessation (16, 18, 22, 25, 27–31, 33). Compared to asking patients’ smoking status and discussing risks of tobacco use with patients, dentists and dental students are significantly less confident in terms of discussing e-cigarettes with patients (31), prescribing cessation medication (18, 27, 31) and arranging follow-up visits for smoking patients (25). Smoking cessation knowledge was assessed in several studies, and most respondents did not possess complete knowledge towards smoking cessation, such as details of 5As, the use (i.e., mechanism, forms, dosage, prescription, side effects) of nicotine replacement therapy (27). In practical terms, a considerable portion of them was not aware of smoking cessation protocol or guideline in the surveyed region or institution (16, 17, 22, 24, 27), and the referral routes of smoking patients (18, 22).

For both dentists with work experience and dental students, there are consistent findings of perceived barriers to carry out smoking cessation. Top cited obstacles were patients lacking motivation to quit (25, 27), patients resistance to smoking cessation advice (17, 18, 21, 24), inadequate training (19, 30, 31), lack of smoking cessation resources (22), lack of treatment time (16, 23), damaging patient-dentist relationship (15), and lack of reimbursement (26).

## 5. Dental professionals’ smoking cessation practices

To evaluate the clinical practices of tobacco intervention, studies asked dental professionals if they performed corresponding behaviors in the 5 A’s model. A range of surveys indicated dental professionals demonstrated a pattern of declining levels of involvement as the providers moved through the ‘‘5 A’s’ protocol, both in specialists and general practitioners. ‘Ask’ and ‘Advise’ are most frequently carried out interventions (16–19, 22, 27, 28, 30, 31). Ford et al. (22) reported 49.5% dentists agreed that prescribing nicotine replacing products was appropriate, yet only 26.7% could put it in practice for most of the patients (22). This shows patient management beyond dental treatment may pose an extra hurdle for dentists to implement in reality. Table 1 summarizes tobacco intervention using 5As model in clinical dental practices.

**Table 1:** Tobacco intervention using 5As model in clinical dental practices.

5As	Description
Ask	Reported as high as 94% dentists always enquire patient’s smoking status. (19) Commonly, dentists recorded the tobacco use in charting. However, there was less assessment of the type of tobacco patients were using, especially non-cigarette product use (cigar, hookah, pipe, e-cigarette, or cannabis) (31).
Advise	It was rated from 40% (29) to 97% (22) in various studies, with majority of them showing more than half of respondents would advise patients to quit smoking, discuss health risks of smoking and benefits of smoking cessation. (16–23, 27–31) Surprisingly the study from Glenys et al. (25) found only 8% of Australian dental students always advised patients to quit smoking.
Assess	It was reported only 19% (18) to 40% (30, 31) of dentists would assess patients’ readiness and interest to change their tobacco use habit, yet a higher figure of 74% (17) from periodontists in USA was found.
Assist	When being asked ‘Did you assist patients in quitting smoking?’, some studies showed quite high percentage of dentists engaging in ‘Assist’, ranging from 55% (21) to 85% (27). However, if specific action of ‘Assist’ was asked, the percentage dropped significantly. The particular behaviour includes making a quit plan (14% (16) to 31% (31)), setting a quit day for patient (6% (18) to 9% (30)), providing educational materials such as information on quitting strategies, quit lines and referrals (6% (25) to 41.8% (16)), prescribing approved medications (2% (16) to 18% (31)) and nicotine replacing products (10% (30) to 36% (18)).
Arrange	A remarkably lower percentage of dental professionals showed actions in arranging referral (1% (25) to 47% (21)) for patients while 59% (22) would re-evaluate the tobacco use of patients in subsequent visits.

## 6. Factors affecting dental professionals’ practices in smoking cessation

Despite multiple perceived barriers in delivering smoking cessation advice, studies indicated these obstacles are not the most important factor to provide cessation practice. Rather, in statistical analysis, dentists’ own confidence and willingness are the strongest correlating factors to initiate the practice (18, 22, 29, 31). The most associated factor influencing providers’ confidence in turn to be amount of training (12, 15, 17, 18, 33). After receiving formal training, dentists will be more familiar with interventional techniques. They are also more aware of current cessation guideline (17). Possession of this knowledge will subsequently promote their belief and confidence that smoking cessation practice could be effective and helpful to patients (18). Having an increased degree of confidence, trained dentists could be more engaged in clinical management of smoking patients than their non-trained counterparts. Li et al. (15) reported that a trained dentists were 8 times more likely to discourage patients’ tobacco use and 14 times more likely to know the local referral group. Discouragingly, ‘lack of training’ was one of the top cited barriers. Various studies revealed there was low training rate regarding smoking cessation of general practitioners and even specialists such as periodontists and pediatric dentists (15, 17, 20, 23, 28, 33). Rates of respondents who received training of cessation treatment ranges from 9% (23) to 47% (17) which remained a low percentage. Similarly, since not every dental school include didactic teaching on smoking cessation in the syllabus, only 25%–45% (24, 25) of dental students have received teachings or instructions on cessation strategies from their schools. However, types and content of training received by respondents were mentioned in literature. Majority of dentists agreed that the formal training should be incorporated into dental curriculum (19, 20).

Other factors affecting practice of smoking cessation might include (1) the work environment, (2) smoking status of providers and (3) work experience or education level of providers. Government dentists were more likely to carry out smoking cessation as there were written protocols in government clinic for them to follow (15, 19). Solo private dentists are associated with more barriers in delivering smoking cessation (17). Smoking status of dental professionals and work experience of dentists have uncertain effects on practices of smoking cessation. For smoking status, its association with the amount of cessation interventions are inconclusive. On one hand, smoking dentists were reported less involved in delivering of smoking cessation advice (19); on the other hand, smoking dentists in some studies showed a more favourable attitude in smoking cessation (21, 22). Besides, there are studies showing no correlation between tobacco use of dental professionals and their management of smoking patients (17). For work experience or education level, old dentists are more experienced and show better attitudes towards smoking cessation activities (19). In other studies, fresh graduates were more involved since they tend to receive more training, such as motivational interviewing technique (17, 20, 22, 30) and they also demonstrated more frequent practice of asking patients’ smoking status (29). While in dental school, students in more senior years who possessed better knowledge were more likely to view smoking cessation as scope of dental practice (25, 26). No conclusion can be drawn for these cited factors since most studies did not aim at exploring the effect of demographics on dental practice of smoking cessation.

## 7. Patients’ perception of smoking cessation service in dental setting

Patients’ knowledge of impact of smoking was tested in a limited number of articles. Patients understood well the detrimental effects of smoking to systemic health, yet they showed less awareness on the effects of tobacco on oral health (32, 34). The relationship between smoking and periodontal disease, impaired wound healing and oral cancer were not well-perceived by patients, specifically smokers showed lower level of understanding (32, 35). Lung et al. (35) reported only 6% of patients could state how smoking affect the periodontium. Patient’s welcoming attitude towards smoking cessation advice delivered by the dental personnel is a consistent finding in various studies (32, 34, 36–40). It is described that advice from dental practitioners is more favorably delivered than advice within other settings (38). Studies suggested majority of patients expected the dentist to be interested in their smoking status (range from 78% (40) to 89.4% (34)). Dentists are also expected to explain the effects of smoking to systemic and oral health, advise patients to quit if smoking affects their oral health and assist patients to quit. In line with this receptive attitude, only a minority of patients would not appreciate cessation advice from dentists and would show negative feelings towards practice of smoking cessation in dental setting (i.e., Feeling uncomfortable, annoyed, embarrassed) (37). On the contrary, patients had negative views towards past dental experience in relation to lack of advice by the previous dentist (38). This reflects patients’ recognition and acceptance of smoking cessation in dental setting.

## 8. Discussion

According to studies, when compared to medical practitioner, dentists were found to be less active in smoking cessation (17). A study in the Netherlands compared 14 groups of healthcare providers. It was shown that dentists had the lowest advice and referral rate compared to other groups (16, 41). It is thus important to overcome barriers and improve the cessation practice in dental practice since oral health professionals have an indispensable role in prevention of diseases.

In terms of patient barrier, by comparing patients’ and dental professionals’ perception, it seems the latter might have a wrong assumption that patients are absolutely resistant to smoking cessation advice. Patel et al. (17) reported dentists had contradictory view: they stated the cessation intervention was highly successful, at the same time they also believed patients had low acceptance of treatment. Dentists might over-estimate the reluctance of patients. It is reassuring that not many patients would turn a blind eye to the cessation advice from dentists. In fact, most patients did not realize smoking cessation could be performed in dentistry (36). They also did not notice the resources and support to help them quit (39), such as smoking cessation quit line, medications, support groups. Despite not knowing the societal support, most current smokers had tried to quit smoking (34, 37) [mean 3.7 times/person (36)]. Patients might not know the correct way of cutting tobacco use and lose motivation over multiple attempts. As a dental professional, utilizing the personal oral effects (e.g., periodontitis causing tooth loss, drifted teeth, tooth staining, oral cancer) and positive treatment outcome might be useful to motivate patients in smoking cessation (37, 38). Thus, it is important for oral health professionals to initiate discussion on smoking cessation in order to arouse patients’ awareness and knowledge towards the interventions available.

In terms of training, it is widely suggested the dental curriculum should incorporate teaching on smoking cessation. Dental schools may have great influence on dental students’ perception on smoking cessation which in turn affects their future practice of cessation counselling. Given the dental professionals’ knowledge towards effects of smoking is adequate, more focus should be placed on hands-on clinical practice. Based on the review results, details of a specific evidence-based strategy such as the 5A’s model, motivational interviewing, use of nicotine replacing therapy should be highlighted. Cessation protocol, referral routes or any regional recommendations in smoking cessation should also be discussed so it can be integrated into clinical practice. A modified approach to carrying out smoking cessation in healthcare setting called Ask-Advice-Connect (AAC) was proposed to tackle the referral barrier (9, 42). AAC aims at actively connecting patients to smoking cessation treatment and shifting counselling and referral burden from clinicians. In this model, patients are assessed, advised and briefly counselled by primary healthcare providers. Information of patients’ willingness to receive smoking cessation service are sent to Quitline within 24 h and 5 subsequent counselling phone calls are made to reach patients. This resulted 13-fold increase in patient enrollment into smoking cessation treatment than simply giving patient a referral card (Ask-Advice-Refer model). Limited number of studies are conducted since this level of smoking cessation support requires substantial institutional collaboration and well-established system in Quitline service. Future research could investigate if AAC could yield better abstinence outcome in dental setting.

There are few limitations of using results from cross-sectional studies. Firstly, there is no standardized assessment method of this topic. The data has to be interpreted cautiously since every single study varied much in the study method: study target, demographics of study groups, design and questions in questionnaires. For example, different studies had divergent ways to evaluate the ‘practice of smoking cessation’. Some evaluated generally whether dentists help patients quit, while some assessed specifically if dentists assist patients by formulating quit plan, prescribing medications, referring to specialists or Quitline. Similarly, when judging whether dentists are well-informed to carry out cessation, only a few studies tested the knowledge level of practitioners while most asked the self-perception of knowledge level. More favourable response might be generated by self-reported answers. The wide variation among studies limits the validity and viability of comparison of data across studies or across time.

Secondly, the results from cross-sectional studies might not be adequately representative. Most respondents were dentists or specialists practicing in one region or dental students from one dental school. Dental professionals who are more interested in this topic may be more likely to complete the survey, leading to an over-optimistic response. Thus, the results obtained might not be generalized. In addition, there is a lack of longitudinal comparison of the change in attitude, confidence and practice of smoking cessations.

Future research could direct on formulating a core questionnaire to assess dental professional’s role, attitude and practices in smoking cessation counselling. A standard example is The Global Health Professions Student Survey which was released in 2004 by the WHO to collect data from third-year students pursuing advanced degrees in dentistry, medicine, nursing and pharmacy. The survey was designed to assess tobacco use, views on smoking cessation counselling of students in different countries. Modifications could be made onto this survey to allow standardization of data collection and analysis, comparison of data across studies or time, on the same ground. Such that, a more precise and comprehensive understanding of the situation can be obtained.

## 9. Conclusion

This article identifies attitudes, preparedness in terms of confidence, knowledge, and practices of smoking cessation services of dentists and dental students. In general the professional role and responsibility to manage tobacco-using patients are well-perceived. Collective evidence has showed that dental professions could have ample opportunities to knows patient’s smoking history, provide suitable cessation advice, assist patients’ quit attempt and follow up the quitting progress. However, there are several barriers refraining them from helping patients quit smoking with the most important one being the lack of confidence and self-efficacy. By contrast, dental patients may need more assistance in smoking cessation since they may not properly acknowledge the effect of smoking on oral health and the appropriate method to abstain tobacco use. It is believed that with continuous training and education on smoking cessation, dental professionals can be more confident to manage smoking patients. Future research is needed to gain more generalized data from dental practitioners and better assess their attitudes, practices and potential barriers of smoking cessation counselling. This information is crucial and potentially helpful to improve on continuous education of dentists and curriculum in dental schools.
